# Mexican Multicenter Experience of Metastatic Spinal Disease

**DOI:** 10.7759/cureus.58546

**Published:** 2024-04-18

**Authors:** Gervith Reyes Soto, Bernardo Cacho-Díaz, Hugo Vilchis-Sámano, Ignacio Diaz-Sanabria, Beatriz Baeza-Velia, David Ayala-Garduño, Carla García-Ramos, Luis Miguel Rosales-Olivarez, Armando Alpízar-Aguirre, Jeff Natalaja Mukengeshay, Alejandro Reyes-Sánchez, Manuel de Jesus Encarnacion Ramirez, Nicola Montemurro

**Affiliations:** 1 Neurosurgical Oncology, Mexico National Cancer Institute, Mexico City, MEX; 2 Head and Neck Surgery Department, Instituto Nacional de Cancerología, Mexico City, MEX; 3 Spine Surgery, Hospital de Traumatología y Ortopedia “Lomas Verdes”, Mexico City, MEX; 4 Spine Surgery, Instituto Nacional de Rehabilitación “Luis Guillermo Ibarra Ibarra”, Mexico City, MEX; 5 Neurosurgery, Clinique Ngaliema, Kinshasa, COD; 6 Neurological Surgery, Peoples’ Friendship University of Russia, Moscow, RUS; 7 Neurosurgery, Azienda Ospedaliero Universitaria Pisana (AOUP), Pisa, ITA

**Keywords:** spinal metastasis, neurosurgery, multicenter study, solitary metastasis, spinal cord tumor surgery

## Abstract

Background

Spinal metastatic disease is a silent progressive cancer complication with an increasing prevalence worldwide. The spine is the third most common site where solid tumors metastasize. Complications involved in spinal metastasis include root or spinal cord compression, progressing to a declining quality of life as patient autonomy reduces and pain increases. The main objective of this study is to report the incidence of patients and typology of spinal metastases in three reference centers in Mexico.

Methodology

Retrospective cohorts of patients diagnosed with spinal metastases from January 2010 to February 2017 at the National Cancer Institute, National Rehabilitation Institute, and the Traumatology and Orthopedics Hospital “Lomas Verdes” in Mexico City were analyzed.

Results

A total of 326 patients (56% males) with spinal metastases were reported. The mean age was 58.06 ± 14.05 years. The main sources of spinal metastases were tumors of unknown origin in 53 (16.25%) cases, breast cancer in 67 (20.5%) cases, prostate cancer in 59 (18%) cases, myeloma in 24 (7.4%) cases, and lung cancer in 23 (7.1%) cases.

Conclusions

The data obtained in this analysis delivers an updated standpoint on Mexico, providing the opportunity to distinguish the current data from global references. Collecting more epidemiological information for better recording of cancer and its associated complications, as well as further studies on them, is necessary.

## Introduction

The incidence of new cancer cases by 2040 is estimated to be 29.4 million according to the World Health Organization [1] due to the increase in life expectancy and the presence of solid tumors that increase the risk of metastasis to different sites, including the spinal column [2]. The spinal column is the third most common site after the liver and lung for metastases in cancer patients, and the primary tumors that metastasize to the spine include breast, prostate, lymphomas, kidneys, and lung cancer [2]. Spinal metastases are an increasingly frequent complication, along with the development of root or medullar compression as pathological fractures [1,2]. Coleman et al. [2] reported a high prevalence of breast, prostate, and lung cancer, accounting for more than 80% of patients with bone metastases, and the most frequent site for bone metastases was the spine in 87%, followed by the ribs in 77% and pelvis in 67% [2]. About 13% of primary tumors remain unknown [3]. The thoracolumbar region accounts for 70% of all lesions due to vertebral metastasis, the lumbosacral segment accounts for 22%, and the cervical region accounts for just 8% [3]. Up to 60-70% of patient deaths due to a malignant neoplasm have vertebral metastasis at their necropsy, 15-40% present clinically symptomatic disease before death [3], and 10% require surgical management [1]. The natural history of the metastatic spine, in the absence of any medical therapy (chemotherapy or radiotherapy), is a partial or total compression of the spinal cord resulting in spinal injury, which seriously affects the quality and autonomy during life [4]. The morbidities most commonly associated with bone metastasis include pathological fractures, bone pain, and spinal cord compromise [5]. Linked difficulties are further common in osteolytic lesions, as seen in breast cancer and multiple myeloma [6-8].

Vertebral metastasis appears between the fifth and sixth decade of life and the main route of dissemination is hematogenous [1]. Oncological treatment must be offered once a diagnosis of the origin of the primary tumor that has metastasized is obtained [9,10]. Meanwhile, surgical stabilization and decompression are highly recommended for patients with radioresistant tumors in the setting of high-grade spinal compression [11,12]. Part of the problem in national public health is the lack of epidemiological studies to provide current data specifically related to cancer and metastases at the vertebral level [13,14]. Cancer is one of the leading causes of death worldwide, with a high prevalence of complications such as metastasis, diverse sequelae, and a high rate of mortality [15]. It is necessary to conduct analyses to allow a better understanding of the natural history of metastases, obtain statistical data, and improve prevention measures, suitable diagnosis, and timely treatment. Hence, our main objective is to describe patient experiences with spinal metastasis from three reference centers in Mexico.

## Materials and methods

Data source

Three hospitals participated in the database. The study was conducted in accordance with the Declaration of Helsinki and approved by the Ethics Committee of the National Institute of Cancerology Oncology (January/2017). Data were anonymized. Patients were identified by ID codes rather than names, and hospitals were identified by institution ID for privacy reasons The data collected included gender, age at diagnosis of metastases, and affected regions such as cervical, thoracic, lumbar, and thoracolumbar spine. It was considered according to the analyzed bibliography of the most affected sites and a cohort point at 60 years. The pattern of metastasis was evaluated on radiographs, along with the type of cancer and the treatment regimen used (chemotherapy, radiotherapy, or surgery). A retrospective cohort of patients with spinal metastases seen at three referral centers from January 2010 to February 2017 at the National Cancer Institute, National Rehabilitation Institute, and the Traumatology and Orthopedics Hospital “Lomas Verdes” in Mexico City was analyzed. Inclusion criteria were cases selected based on the onset diagnosis of spinal metastasis, including all age groups. Patients with multiple primary cancers were excluded.

Statistical analysis

Continuous variables with normal distribution are presented as means and standard deviation (SD). Non-normally distributed data is presented as the median and interquartile range (IQR). Quantitative variables are presented as numbers and percentages. Associations between variables were assessed using the chi-square test or Mann-Whitney U test depending on the variables. SPSS version 23.0 (IBM Corp., Armonk, NY, USA) and Microsoft Excel (Microsoft Corp., Redmond, WA, USA). The chi-square test was used to test the associations of variables with a significance level of 0.05.

## Results

A total of 326 patients with spinal metastases were reviewed in this study, of whom 189 (56.74%) were male and 136 (43.25%) were female. The mean age of the study group was 58.06 ± 4.05 years at the spinal metastasis diagnosis. Breast cancer was the most common with 67 (20.5%) cases, followed by prostate cancer with 59 (18%) cases, unknown primary tumor with 53 (16.25%) cases, non-specific carcinomas with 25 (7.6) cases, myeloma with 24 (7.4%) cases, lung cancer with 23 (7.1%) cases, lymphoma with 17 (5.2%) cases, kidney cancer with 13 (4%) cases, non-seminomatous germ cell tumors with eight (2.5%) cases, cervical cancer with seven (2.2%) cases, and hepatocellular with five (1.5%) cases. Table 1 shows all the details, and Table 2 shows the report from tumors recognized as non-specific.

**Table 1:** Study population. NSGCTs = non-seminomatous germ cell tumors; MCC = Merkel cell carcinoma

Parameter	Gender, N (%)			Age (years)	
N = 326	Female	Male	Total		
Sex	141 (43.25)	185 (56.74)	326 (100)	Mean	SD
Primary cancer					
Breast cancer	67 (47.5)	-	67 (20.5)	54.22	11.883
Prostate	-	59 (31.9)	59 (18)	67.1	9.83
Multiple myeloma	6 (4.3)	18 (9.7)	24 (7.3)	57.54	9.641
Lung	9 (6.4)	14 (7.6)	23 (7)	59.43	12.688
Lymphoma	2 (1.4)	15 (8.1)	17 (5.2)	46.24	15.806
Renal cancer	6 (4.3)	7 (3.8)	13 (3.9)	65.92	10.92
NSGCTs	1 (0.7)	7 (3.8)	8 (2.4)	29.37	10.35
Cervicouterine	7 (5.0)	-	7 (2.1)	62.29	10.49
Hepatocellular	1 (0.7)	4 (2.2)	5 (1.5)	71.6	14.91
Thyroid	1 (0.7)	3 (1.6)	4 (1.2)	63.5	8.505
Melanoma	3 (2.1)	-	3 (0.9)	39.33	12.22
Synovial sarcoma	1 (0.7)	1 (0.5)	2 (0.6)	66.5	16.263
Epidermoid		2 (1.1)	2 (0.6)	56	15.55
Neuroendocrine	1 (0.7)	1 (0.5)	2 (0.6)	53	11.314
Colon		2 (1.1)	2 (0.6)	39.5	2.121
Others	5 (3.5)	5 (2.7)	10 (6.3)	49.2	10.23
Unknown primary tumor origin	31 (21.9)	47 (25.4)	78 (23.92)	59.12	13.47
Total	141 (100)	185 (100)	326 (100)	-	-

**Table 2:** Tumors recognized as non-specific carcinomas.

Unknown primary tumors: Pathology report	N (%)	Gender (%)	
Non-specific adenocarcinoma	66 (52)	25 (80.64)	41 (87.23)
Metastatic carcinoma	5 (20)	3 (9.67)	2 (4.25)
Low-differentiated carcinoma	5 (20)	2 (6.45)	3 (6.38)
Atypical cells in *desmoplasia*	1 (4)	0	1 (2.12)
Lacunar binucleated eosinophilic cells	1 (4)	1 (3.2)	0
Total	78 (100)	31 (39.74)	47 (60.25)

Stratification according to sex was performed. The tumors with the highest frequency for women were breast cancer in 67 (47.5%) cases, followed by lung in nine (6.4%) cases, cervical in seven (5%) cases, and renal and multiple myeloma in six (4.3%) cases. Meanwhile, for men, the highest frequency reported was prostate cancer in 59 (31.9%) cases, multiple myeloma in 18 (9.7%) cases, lymphoma in 15 (8.1%) cases, and lung in 14 (7.6%) cases. According to the age of presentation of metastasis, breast cancer patients had an average age of 54.22 ± 11.88 years (range = 28-84 years). In the case of patients with lung cancer, the mean age was 59.43 ± 12.68 years (range = 30-79 years). For patients with prostate cancer, the mean age was 67.10 ± 9.83 years old (range = 49-88 years). Stratification was performed by age with a cohort point of 60 years. In the group of patients under 60 years old, the highest frequency was breast cancer with 47 (26.6%) cases, followed by prostate cancer in 20 (11.3%) cases, myeloma multiple in 16 (9%) cases, lymphoma in 12 (6.8%) cases, and lung cancer in 11 (6.2%) cases. Older patients in the age group of 61-89 years presented the following frequency of cancer: prostate in 39 (26.4%) patients, breast in 20 (13.5%) patients, lung in 12 (8.1%) patients, renal in 10 (6.8%) patients, and multiple myeloma in eight (5.4%) patients.

**Figure 1:**
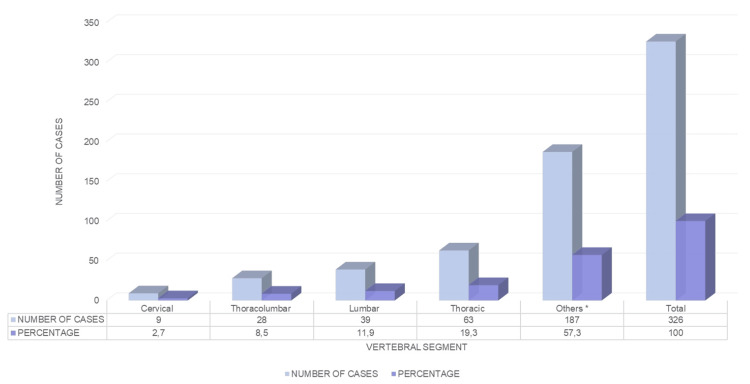
Affected vertebral segments in the study population. Others*: non-contagious segment conditions or with more than two segments involved.

The number of vertebrae affected was a minimum of 1, and a maximum of 13, with an average of 1.82 ± 2.0 vertebrae. Overall, 27% (88/326) of the sample had one or two vertebrae affected, while the rest of the group was dispersed between different numbers of affected vertebrae. The most affected segment by metastasis was the thoracic segment with 63 (19.3%) patients, followed by the lumbar region in 39 (11.3%) patients, thoracolumbar in 28 (8.5%) patients, and cervical in nine (2.7%) patients. The rest of the cases (187 cases) were reported as non-contiguous segments or with the involvement of two or more segments (Figure 1). The predominant pattern of destruction observed on radiographs was lithic in 103 (31.7%) cases, mixed in 49 (15.1%) cases, and burst in 41 (12.6%) cases. Overall, 35 (10.8%) patients underwent palliative surgery, 123 (37.8) patients received chemotherapy management alone, and 141 (43%) patients received radiotherapy management alone.

## Discussion

The current literature fails to characterize the epidemiology of spinal metastasis in Mexico [16-19]. Despite the small sample, this study adds unique knowledge from three hospitals in Mexico, as well as an in-depth analysis not reported previously. These results can support the development of screening programs to detect metastasis in some patients. Training physicians in understanding how these injuries affect expectance and quality of life will improve earlier identification of patients at a heightened risk of development of spinal metastases and lead to early diagnosis and acceptable treatment of these injuries.

The unknown primary tumor was the most common in our series compared with 13% of the unknown primary tumors reported by Witt et al. [2]. Cancer of unknown origin was the most common at 23.9%, with a gap of 10% more. This could be due to multiple factors, including a sufficient quantity of tumor tissue or the lack of availability to perform molecular tests. In Mexico, breast cancer is diagnosed in locally advanced stages (IIb-III) in 55.9% of cases and metastatic stages (IV) in 10.5% of cases, with 64.5% of cases diagnosed between these two stages [14]. There is a high probability of the presence of metastasis such as the spine, liver, and lung already [20-22]. These factors may contribute to the increased rate of unknown-origin tumors. Additionally, the difference between countries with a better socioeconomic level may help identify patients in earlier stages due to a superior implementation of screening and sophisticated tests for diagnosis such as specific molecular tests. This is crucial because the presence of a tumor of unknown origin is associated with a poor prognosis and overall survival of no more than six months [23].

The NOMS decision framework consists of neurologic, oncologic, mechanical, and systemic considerations and incorporates the use of conventional external beam radiation, spinal stereotactic radiosurgery, and minimally invasive and open surgical interventions [24]. A review of radiation oncology and surgical literature that examined the outcomes of treatment of spinal metastatic tumors provided support for the NOMS decision framework [24].

The age group most frequently impacted by spinal metastases is 40 to 70 years. In men, this is associated with a heightened predilection for prostate cancer to cause bone metastases. Prostate cancer is the most frequent in men older than 60 years and has the highest associated mortality rate. In women, breast cancer is the most frequent [16]. In women over the age of 30, the risk of breast cancer increases in parallel and exponentially. In women over 60 years of age, the incidence rate is 104.5 cases per 100,000 women, and it is the most frequent cause of death for women in this age group in Mexico. This means that as their age increases, there is a greater presence of malignant breast tumors and higher mortality associated with them [25]. Overall, 70% of women with advanced cancer will have vertebral metastases [26]. According to the data obtained from our analysis, national and international data can be correlated, which coincide in the most frequent tumors depending on sex and age: breast cancer in women over 60 years of age and prostate cancer in men in the same age group. The age group of 61-89 years presented the following frequency of cancer: prostate at 26.4%, followed by breast at 13.5%, and lung at 8.1%.

The thoracolumbar region accounts for 70% of all lesions due to vertebral metastasis, the lumbosacral segment accounts for 22%, and the cervical region accounts for just 8% [27]. In our study, the most affected segment was the thoracic at 19.4%, followed by the lumbar region at 12%, thoracolumbar at 8.6%, cervical at 2.8%, and the rest were reported as non-contiguous segments or with involvement of two or more segments. We found a significant difference in the number of levels affected by vertebral metastasis, Overall, 60-70% of vertebral metastases were in the thoracolumbar region. In our study, this was in third place at 8.6%, below the involvement of two non-continuous segments or two or more involved vertebral segments that represent more than 57%. This statistical difference is given by a sub-classification in which thoracolumbar tumors were divided into thoracic, lumbar, and thoracolumbar, with the totality of these resulting in 40%, unlike previously reported literature. According to the literature, up to 30% corresponded to lesions in segments that were not contiguous at the time of metastasis diagnosis and may or may not be symptomatic [28-31]. Although we cannot define what caused this variability, it can be suspected that with a later diagnosis of a primary solid tumor, there is a greater probability that metastasis detected at the vertebral level will be present in two or more non-contiguous sites.

Wright et al. [7] conducted a study with 2,148 participants globally and described the primary epidemiological differences in vertebral metastases. In Asian countries, the most prevalent types of vertebral metastases were from the liver (14% vs. >5% in the rest of the world) with a similar incidence of lung metastasis (28% vs. 16%), a lower incidence of breast cancer (6% vs. 21% in the rest of the world), metastases from prostate neoplasms were less common in Asia at 5% and North America at 7% compared to the rest of Europe at 18%. Sarcomas had a higher prevalence in the United Kingdom and the United States at 3% and 5%, respectively, compared to the rest of Europe at 1%. The age of manifestation was similar in all continents with an average of 58-62 years.

The therapeutic trend globally is an isolated posterior approach, preferred 77% of the time in North America compared to 94% in Asia. The combination of anterior and posterior approaches is the second preferred approach globally, with a similar number of preoperative embolizations in 22% of the cases. The percentage of tumor resection significantly varies among Asia, Europe, the United States, and the United Kingdom. Meanwhile, in Mexico, there are no studies correlating the types of primary cancer with vertebral metastasis. National and international epidemiological data record the most frequent types of malignant tumors in the Mexican population according to sex, age, and the most frequent type of tumor [8]. According to the Global Cancer Observatory 2020, in Mexico, the most frequent cancers were breast (23%), followed by prostate (22%) and colorectal (8%) [8]. The National Institute of Statistics and Geography in 2021 reported 90,603 deaths due to malignant tumors, with a gender distribution of 51% in men and 49% in women. Mexico City, Sonora, Chihuahua, Morelos, Veracruz, and Colima were the states with the highest death rates due to malignant tumors in the country [7].

Bone cancer can be classified as osteolytic or osteoblastic according to the characteristics of the radiographic appearance of the lesion. It depends on the type of lesion that predominates, such as lysis, sclerotic, or mixed [32]. When osteoclastic lesions are dominant, they favor the emergence of lytic features, frequent in tumors such as lung or multiple myeloma. The increased osteoblastic activity forms dense sclerotic lesions, usually found in prostate metastases [33]. Both osteoblastic and osteoblastic processes are increasing and there may be mixed manifestations, as seen in metastatic breast cancer [34,35]. The predominant pattern observed in the analysis of destruction observed on radiographs was lithic in 31.7%, mixed in 15.1%, burst in 12.6%, and indeterminate in 40.6%. This coincides largely with the data obtained with the type of lesion identified in the most frequent primary tumors, except for the indeterminate type. The reason why the type of injury could not be identified could likely be operator-dependent viewing of the radiographs [36-38].

Radiotherapy is the central treatment proposed for patients with spinal metastasis except for those with unsatisfactory prognosis, low life expectancy, or primary hematological cancer [34]. The major goal is pain control and improvement or prevention of neurological compromise. The goal is to reduce pain by up to 60% to 70% partially and up to 25% complete pain resolution for up to three to four months [39]. Multiple treatment regimens have been described, but they must be approached according to the radiosensitivity of the tumor, accessibility to treatment, and the risk-benefit ratio due to the increased probability of impairing healthy tissue, including the bone marrow [40]. Surgery, unlike radiotherapy, does not have a curative purpose or reduce pain. The objective of surgery is to provide stability to the spine, spinal cord decompression, and as an adjuvant in radiosurgery treatment [41]. In patients with poor survival, a high risk of undergoing surgery, or by patients’ own decision, palliative management can be offered that includes algology, psychiatry, and physical rehabilitation [42,43]. Overall, 10.8% underwent palliative surgery during the hospital stay, 37.8% received chemotherapy management, and 43% received radiotherapy management.

A modern framework for the treatment of metastatic spine tumors must emphasize durable tumor control while minimizing treatment-related morbidity. Effective pharmacologic, radiation, and surgical treatment options must be considered to achieve this goal. NOMS provides a framework that facilitates decision-making and can optimize patient care [24].

Regarding treatment decisions, there is no specific treatment or therapy. The choice of therapy should be based on the patient’s condition, the presence of root or medullar compression and spinal cord instability, severity, and the availability of hospital aids. Multidisciplinary management benefits patients and offers the best diagnostic tools and treatment types such as three-dimensional (3D) printing. This allows for the creation of patient-specific anatomical models based on their imaging data, as well as the use of telemedicine [44-46]. These models provide a tangible representation of the patient’s spine, including the metastatic lesions, enabling surgeons to understand the complex anatomy and plan the surgical approach more effectively. Surgeons can use these models to simulate different surgical approaches and techniques before the actual surgery [47]. This preparation can lead to a reduction in operative time and potentially lower the risk of complications. 3D printing can be used to create custom surgical instruments and implants tailored to the patient’s anatomy. This ensures a better fit and may improve the stability of the surgical construct [48].

Study limitations

The main limitation of the study was the small sample size that could be expanded to reach a higher significance level, as well as the fact that it was a single-center study. Further studies could provide a more comprehensive understanding of disease prognosis.

## Conclusions

The accelerated demographic transition, as well as the epidemiological and nutritional transition in the Mexican population, makes the population susceptible to various risk factors for cancer. Diagnosis in advanced clinical stages is another challenge in Mexico. The data obtained in this study delivers an updated standpoint on Mexico, providing the opportunity to distinguish the current data from the global references. Breast cancer and lung cancer metastases appear to be the most affected tumors in females, while prostate cancer, multiple myeloma, and lymphoma localizations appear to be the most affected tumors in males. In addition, a high rate of tumors of unknown primary origin was reported. More epidemiological information and further studies are needed for better recording of cancer and its associated complications.

## References

[1] Torre-González DM De, Aguilar-Araiza MA, Ávila-Fuentes DN (2013). Tumores metastásicos a la columna vertebral. Rev Hosp Jua Mex.

[2] Coleman RE, Croucher PI, Padhani AR (2020). Bone metastases. Nat Rev Dis Primers.

[3] Van den Brande R, Cornips EM, Peeters M, Ost P, Billiet C, Van de Kelft E (2022). Epidemiology of spinal metastases, metastatic epidural spinal cord compression and pathologic vertebral compression fractures in patients with solid tumors: a systematic review. J Bone Oncol.

[4] Witt D, Jaque I, Sepúlveda M (2020). Enfermedad metastásica de la columna vertebral / Metastatic spine disease. Rev Méd Clín Condes.

[5] Barzilai O, Boriani S, Fisher CG (2019). Essential concepts for the management of metastatic spine disease: what the surgeon should know and practice. Global Spine J.

[6] Ryan C, Stoltzfus KC, Horn S (2022). Epidemiology of bone metastases. Bone.

[7] Wright E, Ricciardi F, Arts M (2018). Metastatic spine tumor epidemiology: comparison of trends in surgery across two decades and three continents. World Neurosurg.

[8] INEGI INEGI (2021). Estadísticas a Propósito del Día Internacional contra la Corrupción. https://www.inegi.org.mx/app/saladeprensa/noticia.html?id=8674.

[9] (2024). International Agency for Research on Cancer. Population factsheets. https://gco.iarc.fr/today/en/fact-sheets-populations.

[10] Bilsky MH, Laufer I, Fourney DR (2010). Reliability analysis of the epidural spinal cord compression scale. J Neurosurg Spine.

[11] Yamada Y, Katsoulakis E, Laufer I (2017). The impact of histology and delivered dose on local control of spinal metastases treated with stereotactic radiosurgery. Neurosurg Focus.

[12] Zhou X, Cui H, He Y, Qiu G, Zhou D, Liu Y (2019). Treatment of spinal metastases with epidural cord compression through corpectomy and reconstruction via the traditional open approach versus the mini-open approach: a multicenter retrospective study. J Oncol.

[13] Barzilai O, Amato MK, McLaughlin L (2018). Hybrid surgery-radiosurgery therapy for metastatic epidural spinal cord compression: a prospective evaluation using patient-reported outcomes. Neurooncol Pract.

[14] Alvarez Aquino A, Ramirez MJ, Bozkurt I (2022). Treatment of intracranial tumors with stereotactic radiosurgery: short-term results from Cuba. Cureus.

[15] Reyes Soto G, Cacho-Díaza B, Bravo-Reynab C (2023). Prognostic factors associated with overall survival in breast cancer patients with metastatic spinal disease. Cureus.

[16] Sugita S, Ogiso S, Fujiwara M, Morita E, Koyama T, Hozumi T (2023). The outcome of molecularly targeted therapy after surgical treatment of spinal metastasis. J Clin Med.

[17] Cárdenas-Sánchez J, Valle-Solís AAE, Arce-Salinas C (2019). Consenso Mexicano sobre diagnóstico y tratamiento del cáncer mamario. Mexican J Oncol.

[18] Losa F, Soler G, Casado A (2018). SEOM clinical guideline on unknown primary cancer (2017). Clin Transl Oncol.

[19] Landriel F, Lichtenberger FP, Ulloque-Caamaño L, Mosquera C, Aineseder M, Perotti JM, Hem S (2023). Measuring the delay in the referral of unstable vertebral metastasis to the spine surgeon: a retrospective study in a Latin American institution. Neurol India.

[20] Plancarte R, Guajardo J, Meneses-Garcia A (2014). Clinical benefits of femoroplasty: a nonsurgical alternative for the management of femoral metastases. Pain Physician.

[21] Curtin M, Piggott RP, Murphy EP, Munigangaiah S, Baker JF, McCabe JP, Devitt A (2017). Spinal metastatic disease: a review of the role of the multidisciplinary team. Orthop Surg.

[22] Niglas M, Tseng CL, Dea N, Chang E, Lo S, Sahgal A (2020). Spinal cord compression. Abeloff's Clinical Oncology (Sixth Edition).

[23] Nurmukhametov R, Enelis B, Bernard E (2022). Thoracic percutaneous vertebroplasty for the treatment of vertebral hemangioma in a patient with Forestier’s disease: a case report. Cureus.

[24] Laufer I, Rubin DG, Lis E, Cox BW, Stubblefield MD, Yamada Y, Bilsky MH (2013). The NOMS framework: approach to the treatment of spinal metastatic tumors. Oncologist.

[25] Spratt DE, Beeler WH, de Moraes FY (2017). An integrated multidisciplinary algorithm for the management of spinal metastases: an International Spine Oncology Consortium report. Lancet Oncol.

[26] Reyes Soto G, Ovalle Torres CS, Perez Terrazas J (2023). Multiple myeloma treatment challenges: a case report of vertebral artery pseudoaneurysm complicating occipitocervical arthrodesis and a review of the literature. Cureus.

[27] Ran N, Li W, Zhang R (2023). Autologous exosome facilitates load and target delivery of bioactive peptides to repair spinal cord injury. Bioact Mater.

[28] Meyer M, Farah K, Aurélie T, Graillon T, Dufour H, Fuentes S (2023). Management of spinal metastasis by minimally invasive surgical techniques: surgical principles and indications-a literature review. J Clin Med.

[29] Amelot A, Terrier LM, Cognacq G (2023). Natural history of spinal cord metastasis from brain glioblastomas. J Neurooncol.

[30] Nourbakhsh A, Harrison K (2023). Use of steroids in spine surgery. J Am Acad Orthop Surg.

[31] Li H, Zhao Y, Ma T, Shao H, Wang T, Jin S, Liu Z (2023). Radiotherapy for extensive-stage small-cell lung cancer in the immunotherapy era. Front Immunol.

[32] Pennington Z, Porras JL, Larry Lo SF, Sciubba DM (2023). International variability in spinal metastasis treatment: a survey of the AO Spine community. Global Spine J.

[33] Zhou JJ, Farber SH, de Andrada Pereira B (2024). Metastasis of intracranial meningioma to the osseous spine: systematic literature review and case report. World Neurosurg.

[34] Takei D, Tagami K (2023). Management of cancer pain due to bone metastasis. J Bone Miner Metab.

[35] Du X, Wei H, Zhang B (2023). Molecular mechanisms of osteosarcoma metastasis and possible treatment opportunities. Front Oncol.

[36] Solomon BJ, Bauer TM, Mok TS (2023). Efficacy and safety of first-line lorlatinib versus crizotinib in patients with advanced, ALK-positive non-small-cell lung cancer: updated analysis of data from the phase 3, randomised, open-label CROWN study. Lancet Respir Med.

[37] van den Bent MJ, Geurts M, French PJ, Smits M, Capper D, Bromberg JE, Chang SM (2023). Primary brain tumours in adults. Lancet.

[38] Nie Y, Ying B, Lu Z, Sun T, Sun G (2023). Predicting survival and prognosis of postoperative breast cancer brain metastasis: a population-based retrospective analysis. Chin Med J (Engl).

[39] Nardone V, Romeo C, D'Ippolito E (2023). The role of brain radiotherapy for EGFR- and ALK-positive non-small-cell lung cancer with brain metastases: a review. Radiol Med.

[40] Lee SF, Yip PL, Chan OL, Lee VW, Wong A, Choi HC (2023). Brain metastasis growth kinetics: a novel prognosticator for stereotactic radiotherapy. Clin Oncol (R Coll Radiol).

[41] Han J, Zhao Z, Wan D (2023). Editorial: potential effects and mechanisms of bone homeostasis on tumor bone metastasis. Front Endocrinol (Lausanne).

[42] Ma X, Zhao Y, Zhao J, Wu H, Feng H (2023). Percutaneous pedicle screw fixation combined with percutaneous vertebroplasty for the treatment of thoracic and lumbar metastatic tumors. J Clin Transl Res.

[43] Amelot A, Terrier LM, Le Nail LR (2023). Spine metastasis: patients with poor performance status (ECOG) could benefit from palliative surgical care! A prospective cohort study. Spine (Phila Pa 1976).

[44] Hernandez CL, Díaz SM, Nurmukhametov R, Goncharov E, Ramirez MJ, Bozkurt I, Ramirez Pena IJ (2022). A case report of a sacral giant cell tumor treated with block resection and lumbo-pelvic fixation. Cureus.

[45] Ramirez MJ, Nurmukhametov R, Bernard E, Peralta I, Efe IE (2022). A low-cost three-dimensional printed retractor for transforaminal lumbar interbody fusion. Cureus.

[46] Montemurro N (2022). Telemedicine: could it represent a new problem for spine surgeons to solve?. Global Spine J.

[47] Ramirez MJ, Nurmukhametov R, Musa G (2022). Three-dimensional plastic modeling on bone frames for cost-effective neuroanatomy teaching. Cureus.

[48] Uhl JF, Sufianov A, Ruiz C (2023). The use of 3D printed models for surgical simulation of cranioplasty in craniosynostosis as training and education. Brain Sci.

